# Commitment and Attachment to Pet and Emotional Well-Being Among Low-Income Pet Owners

**DOI:** 10.1093/geroni/igaa057.376

**Published:** 2020-12-16

**Authors:** Ke Li, Fengyan Tang, Mary Rauktis

**Affiliations:** 1 University of Pittsburgh, Pittsburgh, Pennsylvania, United States; 2 social work, Pittsburgh, Pennsylvania, United States

## Abstract

Pets play an important role in older adults’ lives, as people treat pets as their companion and family members. Owning a pet has been believed to be beneficial; however, previous literature demonstrated mixed results of the effects of pet on people’s well-being. Using data collected from 392 food pantry users in Pittsburgh, this study examined the relationships of pet ownership, attachment and commitment with emotional well-being, and investigated whether sociodemographic profiles conditioned those relationships. emotional well-being was assessed by global mental health, positive functioning, perceived negative feelings, and perceived positive feelings. Commitment to pet was measured by a 10-item scale adapted from the Miller-Rada Scale about the likelihood of giving up pet under various difficult circumstances. Attachment to pet was measured by the 23-item Lexington Attachment to Pets Scale about emotional ties to pet. About two-thirds of respondents (66%) were pet owners. Multiple regression analysis showed that pet owners perceived fewer positive feelings (e.g. happy, joyful) than non-pet owners. However, among pet owners, a higher level of pet attachment was associated with more positive feelings. Gender and education significantly moderated the effects of pet ownership on emotional well-being, as male and employed respondents were more likely to benefit from owning a pet. Moreover, the positive effects of attachment and commitment to pet were stronger among respondents with higher levels of education or in the labor force. future studies need to investigate how to promote the benefits of pet companion and address the challenges faced by low-income pet owners.

